# Patient factors associated with receipt of psychological and pharmacological treatments among individuals with common mental disorders in a Swedish primary care setting

**DOI:** 10.1192/bjo.2023.8

**Published:** 2023-02-28

**Authors:** Alexis E. Cullen, Elin Lindsäter, Syed Rahman, Heidi Taipale, Antti Tanskanen, Ellenor Mittendorfer-Rutz, Magnus Helgesson

**Affiliations:** Department of Clinical Neuroscience, Division of Insurance Medicine, Karolinska Institutet, Stockholm, Sweden; and Department of Psychosis Studies, Institute of Psychiatry, Psychology & Neuroscience, King's College London, UK; Department of Clinical Neuroscience, Division of Psychology, Karolinska Institutet, Stockholm, Sweden; Department of Clinical Neuroscience, Division of Insurance Medicine, Karolinska Institutet, Stockholm, Sweden; Department of Clinical Neuroscience, Division of Insurance Medicine, Karolinska Institutet, Stockholm, Sweden; Department of Forensic Psychiatry, University of Eastern Finland, Niuvanniemi Hospital, Kuopio, Finland; and School of Pharmacy, University of Eastern Finland, Kuopio, Finland; Department of Forensic Psychiatry, University of Eastern Finland, Niuvanniemi Hospital, Kuopio, Finland; Department of Clinical Neuroscience, Division of Insurance Medicine, Karolinska Institutet, Stockholm, Sweden; and Department of Public Health and Caring Sciences, Health Equity and Working Life, Uppsala University, Uppsala, Sweden

**Keywords:** Psychiatric disorders, health inequalities, medication, psychotherapy, healthcare

## Abstract

**Background:**

Psychological and pharmacological therapies are the recommended first-line treatments for common mental disorders (CMDs) but may not be universally accessible or utilised.

**Aims:**

To determine the extent to which primary care patients with CMDs receive treatment and the impact of sociodemographic, work-related and clinical factors on treatment receipt.

**Method:**

National registers were used to identify all Stockholm County residents aged 19–64 years who had received at least one CMD diagnosis (depression, anxiety, stress-related) in primary care between 2014 and 2018. Individuals were followed from the date of their first observed CMD diagnosis until the end of 2019 to determine treatment receipt. Associations between patient factors and treatment group were examined using multinomial logistic regression.

**Results:**

Among 223 271 individuals with CMDs, 30.6% received pharmacotherapy only, 16.5% received psychological therapy only, 43.1% received both and 9.8% had no treatment. The odds of receiving any treatment were lower among males (odds ratio (OR) range = 0.76 to 0.92, 95% CI_[minimum, maximum]_ 0.74 to 0.95), individuals born outside of Sweden (OR range = 0.67 to 0.93, 95% CI_[minimum, maximum]_ 0.65 to 0.99) and those with stress-related disorders only (OR range = 0.21 to 0.51, 95% CI_[minimum, maximum]_ 0.20 to 0.53). Among the patient factors examined, CMD diagnostic group, prior treatment in secondary psychiatric care and age made the largest contributions to the model (R^2^ difference: 16.05%, 1.72% and 1.61%, respectively).

**Conclusions:**

Although over 90% of primary care patients with CMDs received pharmacological and/or psychological therapy, specific patient groups were less likely to receive treatment.

Common mental disorders (CMDs), including depression, anxiety and stress-related disorders, affect around 30% of the world's population at some point during life.^1^ These disorders are extremely disabling, with depression and anxiety both ranked within the top 15 most burdensome diseases for individuals aged 25–49 years.^2^ Psychological and pharmacological therapies, which have been shown to be effective in reducing target symptoms in individuals with depression, anxiety, obsessive–compulsive disorder and post-traumatic stress disorder (albeit with small-to-moderate effect sizes and substantial heterogeneity),^3^ are the recommended first-line treatments for CMDs in many countries.^4–11^However, community-based surveys have identified a substantial ‘treatment gap’ (defined as the proportion of individuals who require treatment but do not receive it) among individuals with CMDs. In a 2004 review of these studies, 50–60% of individuals meeting major depression, generalised anxiety disorder and obsessive–compulsive disorder criteria had not received medical or professional services.^12^ More recent population surveys conducted in the UK have reported similar treatment gaps (ranging from 26% to 76%) among those with CMDs.^13–16^ Such surveys have shown that factors including age, gender, ethnic minority and migrant status, socioeconomic disadvantage (e.g. lower education levels, unemployment, low income and receipt of government pensions), type of living area and diagnosis are associated with the likelihood of accessing psychological and pharmacological therapies.^15–21^ However, findings have been inconsistent across studies, many of which did not restrict analyses to individuals meeting CMD criteria (instead controlling for symptom presence and/or severity). Moreover, as such studies include members of the general population, these treatment gaps capture healthcare access (i.e. the extent to which an individual is able and/or willing to access healthcare services in general)^22^ and utilisation of these treatments (i.e. receipt of specific treatments offered by healthcare providers).

## Treatment utilisation in healthcare settings

Identifying factors associated with treatment utilisation is particularly important, as healthcare providers can play a crucial part in developing strategies to ensure equity of treatment provision.^16^ Given that the vast majority of individuals who present to healthcare services with CMDs are treated exclusively within primary care,^23^ it is important to determine the extent to which these patients receive recommended first-line treatments. Previous studies of primary care patients with CMDs have reported highly variable rates of treatment receipt (range: 36–82%).^23–28^ This variation appears to be partially attributable to CMD diagnostic group: for example, two studies using the same UK primary care sample observed that pharmacological treatments (most commonly antidepressants) were received by 82% and 63% of those with depressive and anxiety disorders, respectively, within 3 months of their first diagnosis.^24,25^ Similarly, a Swedish register study observed that antidepressants were used by 47–79% of primary care patients with CMDs (depression, anxiety, obsessive–compulsive and adjustment disorders), whereas anxiolytics were used by 43–63%,^23^ with the lowest rates of pharmacotherapy treatment (irrespective of type) found in adjustment disorder patients. Treatment receipt rates also vary by treatment modality, with several studies showing that individuals with CMDs are more likely to receive pharmacotherapy than psychological therapies.^27–29^ Whether this differs by CMD diagnostic group is unclear. Consistent with the general population surveys described above, several sociodemographic factors (including age, sex, education, marital status and ethnic minority status) have been reported to be associated with treatment receipt in primary care samples.^26–30^ However, findings have been inconsistent, potentially owing to differences in study methodology, treatment modality and patient factors examined. Indeed, the largest studies in this field have typically used a single healthcare register (without linkage to other registers providing important confounding variables) and have examined only one treatment modality (most commonly pharmacotherapy).

To address these knowledge gaps, the present study used administrative healthcare data from Region Stockholm's VAL database^31^ to examine receipt of first-line recommended treatments among individuals diagnosed with CMDs in primary care. A major strength of the VAL database, which contains pseudonymised individual-level data for visits to primary care clinics in Stockholm County, is that it captures delivery of both systematic (e.g. cognitive–behavioural therapy) and non-systematic (e.g. counselling) psychological therapies and multiple diagnoses.^32^ Moreover, these data can be linked to the extensive national registers available in Sweden,^33^ which provide detailed, high-quality information for a range of factors, including sociodemographic characteristics, medication dispensations, use of secondary healthcare services, employment and work disability. We aimed to determine the proportion of individuals with CMDs who received psychological and/or pharmacological treatments and investigate whether patient sociodemographic, work-related and clinical factors were associated with treatment receipt in this setting.

## Method

### Data sources

Data were obtained from the national registers collected by various Swedish health and social insurance agencies, linked by the (pseudonymised) unique personal identification number assigned to all Swedish residents at birth or migration. According to current Swedish regulations, the use of national data for research purposes does not require informed consent from individuals whose data are held in these registers.^34^ The Longitudinal Integrated Database for Health Insurance and Labour Market Studies (LISA),^35^ maintained by Statistics Sweden, was used to identify the source population and obtain sociodemographic variables and unemployment. LISA has collated annual data for the entire adult population (age ≥16 years) of Sweden since 1990; data for the years 2013–2018 were used to enable determination of sociodemographic characteristics in the year prior to cohort entry for the selected sample (who could enter the cohort between 2014 and 2018). Detailed region-of-birth data (reported in Supplementary Material only, available at https://doi.org/10.1192/bjo.2023.8) were obtained from the Longitudinal Database for Integration Studies (STATIV) register,^36^ developed by Statistics Sweden and the Swedish Integration Board, available from 1997 to 2006. The VAL database,^37^ administered by Region Stockholm, was used to determine diagnoses in primary care, defined according to the ICD-10,^38^ and receipt of psychological therapy. The VAL database includes data on primary care contacts (date, healthcare professional and action code) for all public clinics and most privately owned and/or operated clinics from 2003, with diagnoses included since 2014.^39^ The present study used data from 2014 to 2019 (the first available year with full coverage of diagnoses to the most recently available year). Medication dispensations (Anatomical Therapeutic Chemical (ATC) code and date when prescribed) between 2014 and 2019 were obtained from the Prescribed Drug Register (PDR, administered by the National Board of Health and Welfare),^40^ which includes all prescribed and dispensed medications (except for those administered in hospital) since 2005. Treatment in secondary psychiatric care (date and ICD-10 diagnosis) for the years 2009–2019 was obtained from the National Patient Register (NPR, National Board of Health and Welfare),^41,42^ which has captured all in-patient and specialist out-patient care since 1987 and 2001, respectively. We determined work disability (periods of sickness absence and disability pension) for the years 2013–2018 using the Micro-Data for Analyses of Social Insurance (MiDAS, Swedish Social Insurance Agency)^43^ and death during the study period (2014–2019) using the Cause of Death Register (National Board of Health and Welfare).^44^

### Study design and source population

This population-based cohort study included all individuals in LISA who were resident in Stockholm County for every year between 2014 and 2019; those who moved out of the area or died during this period were excluded. Around one-fifth of the population of Sweden resides in this region, which includes the capital city of Stockholm, several other cities and towns and large rural areas.^45^ Using VAL, we identified individuals who between 1 January 2014 and 31 December 2018 had at least one recorded diagnosis (up to eight can be assigned per visit, with no hierarchy) of depression (ICD-10: F32–F39), anxiety (ICD-10: F40–F42) or stress-related disorder (ICD-10: F43). Individuals entered the study on the date of their first CMD diagnosis within this period. We then excluded individuals who were aged <19 or >64 years during the year of cohort entry and those who had any diagnosis of severe mental illness (bipolar disorder, schizophrenia or other psychotic disorder; ICD-10: F30–F31 and F20–F29) or organic mental disorder (ICD-10: F00–F09) recorded in VAL or NPR at any point prior to cohort entry. Individuals were followed until the end of 2019 (minimum follow-up of 1 year) to determine the type of treatment received.

### Treatment definitions

Individuals were categorised according to the type of treatment received during the follow-up period: no treatment versus pharmacotherapy only versus psychological therapy only versus both. Pharmacological treatments considered here were dispensations of antidepressants (ATC code: N06A), anxiolytics (ATC code: N05B), or hypnotics and sedatives (ATC code: N05C and R06AD01), as recorded in the PDR. Psychological therapies (identified using VAL) were defined as any visit with a systematic psychological therapy action code (see Supplementary Material) or any other visit where the healthcare provider was a psychologist, psychotherapist or ‘curator’ (social workers with specialist training in administering psychological therapies). Given that these pharmacological and psychological therapies can be used to treat other mental and somatic disorders, we included only those treatments that occurred proximally to a CMD diagnosis in VAL (up to 1 month before and 1 year after any CMD diagnosis recorded in VAL). Owing to concerns regarding misclassification (e.g. that CMD diagnoses may have been present but not recorded by primary care clinicians at the time when treatment was received), we excluded individuals who only received these treatments outside this 13 month timeframe rather than assigning them to the no-treatment group.

### Predictor variables

Age in years (categorised as 20–25 versus 26–35 versus 36–45 versus 46–55 versus 56–65) was determined for the calendar year of cohort entry. The following sociodemographic predictors were measured on 31 December in the year prior to cohort entry: gender, region of birth (Sweden versus countries within the European Union between 2004 and 2006 (AKA EU-25 countries) versus the rest of the world), family situation (married or cohabiting without children living at home versus married or cohabiting with children living at home versus single without children living at home versus single with children living at home); type of living area (inner city versus rural area), level of education (low, 0–9 years versus medium, 10–12 years versus high, >12 years).

Work-related measures included the number of days of full-time unemployment, measured during the calendar year prior to cohort entry (none versus 1–180 days versus >180 days), and work disability (sickness absence and disability pension), which were obtained for the year (365 days) prior to cohort entry. Individuals in Sweden are entitled to income-related levels of unemployment benefit (from age 16 years) or basic levels (if aged ≥20 years and having no recent job income) when registered as a job seeker at the Swedish Public Employment Service. All individuals aged 16 years or older with an income above a certain level are eligible to receive sickness benefits; payments are covered by the employer for the first 14 days, and thus only periods exceeding 14 days are covered by the Social Insurance Agency and captured in the MiDAS database. Permanent disability pension in Sweden can be granted to individuals aged 30–64 years, whereas individuals aged 19–29 years can receive time-restricted disability pension if work capacity is reduced or compulsory education is not completed. Both sickness absence and disability pension payments can be granted at full-time or part-time level. In the present study, we calculated the net days (the length of the period × extent of the period) for both, such that 30 days of half-time sickness absence payment was converted to 15 net days. These net days were then used to derive categorical variables indexing sickness absence (none versus 1–90 days versus >90 days) and disability pension (none versus any).

For the clinical predictors, we derived a seven-level, mutually exclusive, categorical variable capturing CMD diagnoses assigned in primary care (anxiety only versus depression only versus stress-related only versus depression + anxiety versus depression + stress-related versus anxiety + stress-related versus depression + anxiety + stress-related). As our intention was to determine whether CMD diagnostic group was associated with type of treatment received, for individuals treated with pharmacotherapy and/or psychological therapy, we included only those diagnoses that were recorded between the date of cohort entry and the last observed treatment date; for individuals who received no treatment, all CMD diagnoses recorded during the study period were included. The following clinical factors were measured during the year prior to and year following cohort entry (cohort entry date ± 365 days) with binary variables (none versus any) created for each variable: (a) any other (non-CMD) mental disorder treated in primary care (visits in VAL with any ICD-10 F codes excluding F32–F39 and F40–43); (b) dispensations of any other psychotropic medications (ATC codes N05A, N03AF01, N03AG01, N03AX09, N05AN01, N06B, N07BB, N07BC and N06CA recorded in the PDR); (c) relevant somatic comorbidities treated in primary care for which psychological treatment is indicated as per Region Stockholm primary care guidelines,^46^ namely, endometriosis, irritable bowel syndrome, pain, tinnitus, electrohypersensitivity, obesity, fibromyalgia and post-viral fatigue syndrome/myalgic encephalomyelitis (visits in VAL with ICD-10 codes N80, K58, R52, H93.1, W90, E66, M79.7, G93.3); and (d) any treatment for suicide attempt (intentional self-harm and events of undetermined intent) in primary or secondary care (visits in VAL or NPR with any ICD-10 X60-X84 or Y10-Y34 codes). To capture prior mental disorders that reached sufficient severity to warrant treatment in secondary care, we created a binary variable (none versus any) for any mental disorder, including CMDs, recorded in the NPR in the 4 years prior to and 1 year following cohort entry.

As all individuals were required to be resident in Stockholm Country for each year between 2014 and 2018, calendar year at cohort entry corresponded to time observed. Based on the rationale that individuals with longer observation periods would have more opportunities to receive treatment, this variable was included as a covariate.

### Statistical methods

Analyses were conducted using R version 4.0.4. Descriptive statistics were derived for all predictor variables. A multinomial logistic regression model was conducted using the R ‘nnet’ package with the ‘multinom’ function to examine associations between sociodemographic, work-related and clinical factors (predictors) and type of treatment received (outcome), where the reference category for the outcome variable was the no-treatment group. All predictor variables were entered into the model simultaneously, such that each was mutually adjusted for every other variable in the model. Likelihood-ratio tests were performed for each predictor to test whether inclusion significantly improved model fit. We also derived the Bayesian information criterion (BIC) value (a measure of model fit that introduces a penalty term for the number of parameters in the model) and Nagelkerke pseudo R^2^ (an approximation of the total variance explained) for the overall model. For each predictor in turn, we calculated the difference between the BIC and R^2^ values derived from the full model and the model without the tested variable included. For predictor variables that showed the greatest contribution to the model (Nagelkerke R^2^ value >1%), effects plots were produced using the R ‘effects’ package to show the predicted probability of outcome group membership for each predictor variable, after adjusting for all other variables.

### Ethics statement

All procedures contributing to this work complied with the ethical standards of the relevant national and institutional committees on human experimentation and with the Helsinki Declaration of 1975, as revised in 2008. All procedures involving human subjects/patients were approved by the Ethical Review Board in Stockholm (DNR: 2007/762-31).

## Results

### Study population

**Fig. 1:**
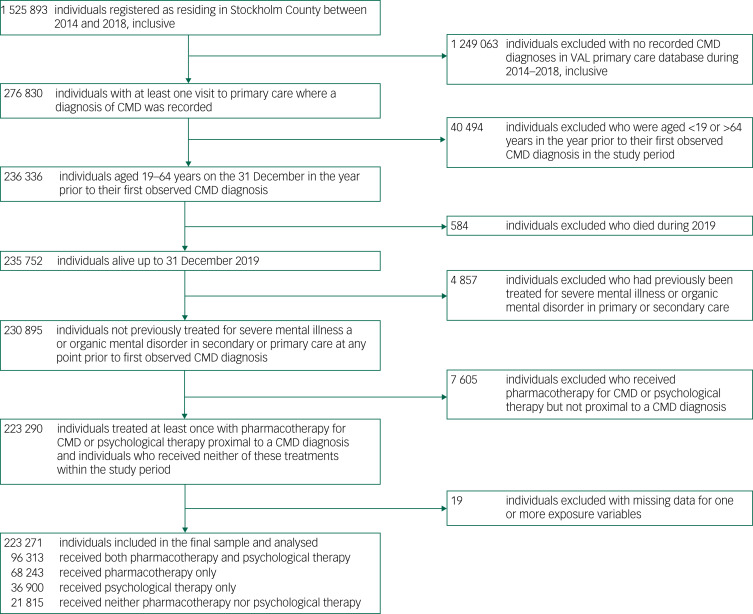
Procedure for deriving study population.

The procedure used to derive the study population is detailed in Fig. 1. Of the 1 525 893 individuals registered as alive and resident in Stockholm County for every calendar year between 2014 and 2018, 276 830 (18.1%) had at least one primary care contact where a diagnosis of CMD was recorded. After excluding individuals based on age, death during 2019, treatment for severe mental illness or organic mental disorder, receipt of pharmacotherapy or psychological therapy that did not occur proximally to a CMD diagnosis and missing data for one or more predictor variables, we included 223 271 individuals who were followed for a median of 4.13 years (range: 1.08–6.00 years).

### Description of treatments

Within the study population, 68 243 (30.6%) individuals received pharmacotherapy only, 36 900 (16.5%) received psychological therapy only, 96 313 (43.1%) had both and 21 815 (9.8%) received no treatment. Of those treated with pharmacotherapy alone or in combination with psychological therapy (*N* = 164 556, 73.7% of the study population), the majority (76.0%) received antidepressant medication, just over one-half (53.6%) received anxiolytics and 51.0% received hypnotics or sedatives (Supplementary Table 2). Among the 133 213 CMD patients (59.7% of the study population) who received psychological therapy alone or in combination with pharmacotherapy, around half (50.8%) had at least one systematic psychological therapy session (predominately CBT), with the rest receiving other therapies administered by a curator, a psychologist or (less commonly) a psychotherapist (Supplementary Table 3).

### Characteristics of the study population

The vast majority of the total study population (Table 1) were aged 26–55 years, with no peak in the age distribution, and two-thirds were female. Most individuals were born in Sweden (75.2%), with Asia (excluding Afghanistan, Iraq, Iran and Syria), Europe (excluding EU15, the Nordic countries and the former Yugoslavia) and Iraq being the three most common regions of birth outside of Sweden (Supplementary Table 4). In terms of work-related factors, fewer than one in ten had been unemployed (for any number of days) in the calendar year prior to cohort entry; just over one in five had received 1–90 days of sickness absence payments in the year (365 days) prior to cohort entry, with far fewer (6.6%) receiving >90 days of payments. Less than 5% had been granted disability pension. Most individuals presented with a single CMD diagnosis (most commonly anxiety); just under a third had diagnoses within two CMD disorder groups, and 7.3% received diagnoses in all three CMD diagnostic groups (see Supplementary Table 5 for specific diagnoses). Only 6% of the sample had received a diagnosis of any other (non-CMD) mental disorder in primary care in the year during or prior to cohort entry, where behavioural syndromes associated with physiological disturbances and physical factors (ICD-10 codes F50–59) were most common (Supplementary Table 6); suicide attempts (treated in primary or secondary care) during this period were also rare (1.5%). However, just under a third (31.7%) had been previously treated for any mental disorder in secondary care, most commonly for CMDs (Supplementary Table 7).

**Table 1:** Characteristics of 223 271 individuals diagnosed with common mental disorders in primary care in Stockholm County by type of treatment received

	No treatment (*N* = 21 815, 9.8%)	Pharmacotherapy only (*N* = 68 243, 30.6%)	Psychological therapy only (*N* = 36 900, 16.5%)	Both (*N* = 96 313, 43.1%)	Total sample (*N* = 223 271)
	*N* (%)	*N* (%)	*N* (%)	*N* (%)	*N* (%)
**Sociodemographic factors**					
Age (years)^a^					
20–25	2396 (11.0)	6011 (8.8)	5010 (13.6)	10 190 (10.6)	23 607 (10.6)
26–35	5487 (25.2)	13 421 (19.7)	11 818 (32.0)	25 302 (26.3)	56 028 (25.1)
36–45	5512 (25.3)	17 199 (25.2)	9656 (26.2)	26 043 (27.0)	58 410 (26.2)
46–55	5031 (23.1)	17 769 (26.0)	6735 (18.3)	22 152 (23.0)	51 687 (23.1)
56–65	3389 (15.5)	13 843 (20.3)	3681 (10.0)	12 626 (13.1)	33 539 (15.0)
Gender, male	7775 (35.6)	25 397 (37.2)	11 215 (30.4)	28 486 (29.6)	72 873 (32.6)
Region of birth					
Sweden	15 599 (71.5)	50 769 (74.4)	28 614 (77.5)	72 885 (75.7)	167 867 (75.2)
EU25 (except Sweden)	1378 (6.3)	4674 (6.8)	2005 (5.4)	5573 (5.8)	13 630 (6.1)
Rest of the world	4838 (22.2)	12 800 (18.8)	6281 (17.0)	17 855 (18.5)	41 774 (18.7)
Family situation^b^					
Married or cohabiting without children	3176 (14.6)	10 529 (15.4)	3731 (10.1)	11 341 (11.8)	28 777 (12.9)
Married or cohabiting with children	7288 (33.4)	18 746 (27.5)	12 365 (33.5)	29 864 (31.0)	68 263 (30.6)
Single without children	9876 (45.3)	33 726 (49.4)	17 894 (48.5)	45 980 (47.7)	107 476 (48.1)
Single with children	1475 (6.8)	5242 (7.7)	2910 (7.9)	9128 (9.5)	18 755 (8.4)
Type of living area, rural^b^	549 (2.5)	1712 (2.5)	726 (2.0)	2562 (2.7)	5549 (2.5)
Level of education^b^					
Low (0–9 years)	2628 (12.0)	10 406 (15.2)	3199 (8.7)	11 243 (11.7)	27 476 (12.3)
Medium (10–12 years)	9207 (42.2)	28 069 (41.1)	14 669 (39.8)	40 961 (42.5)	92 906 (41.6)
High (>12 years)	9980 (45.7)	29 768 (43.6)	19 032 (51.6)	44 109 (45.8)	102 889 (46.1)
**Work-related factors**					
Unemployment (days)^c^					
None	19 832 (90.9)	60 822 (89.1)	33 394 (90.5)	85 324 (88.6)	199 372 (89.3)
1–180 days	1481 (6.8)	5554 (8.1)	2724 (7.4)	8316 (8.6)	18 075 (8.1)
>180 days	502 (2.3)	1867 (2.7)	782 (2.1)	2673 (2.8)	5824 (2.6)
Sickness absence (days)^d^					
0	15 413 (70.7)	49 773 (72.9)	28 142 (76.3)	66 751 (69.3)	160 079 (71.7)
1–90	5667 (26.0)	12 996 (19.0)	7370 (20.0)	22 488 (23.3)	48 521 (21.7)
>90	735 (3.4)	5474 (8.0)	1388 (3.8)	7074 (7.3)	14 671 (6.6)
Received disability pension^d^	505 (2.3)	5980 (8.8)	632 (1.7)	3926 (4.1)	11 043 (4.9)
**Clinical factors**					
CMD diagnosis^e^					
Anxiety	4784 (21.9)	21 571 (31.6)	10 378 (28.1)	18 942 (19.7)	55 675 (24.9)
Depression	2203 (10.1)	16 861 (24.7)	4685 (12.7)	12 076 (12.5)	35 825 (16.0)
Stress-related	13 039 (59.8)	12 497 (18.3)	13 506 (36.6)	11 720 (12.2)	50 762 (22.7)
Depression and anxiety	242 (1.1)	6802 (10.0)	1528 (4.1)	14 593 (15.2)	23 165 (10.4)
Depression and stress-related	575 (2.6)	4042 (5.9)	2026 (5.5)	11 210 (11.6)	17 853 (8.0)
Anxiety and stress-related	887 (4.1)	4380 (6.4)	3834 (10.4)	14 528 (15.1)	23 629 (10.6)
Depression, anxiety and stress-related	85 (0.4)	2090 (3.1)	943 (2.6)	13 244 (13.8)	16 362 (7.3)
Comorbid mental disorder (any)^f^	501 (2.3)	5342 (7.8)	1358 (3.7)	6298 (6.5)	13 499 (6.0)
Comorbid somatic disorder (relevant only)^f^	2161 (9.9)	9971 (14.6)	4330 (11.7)	15 202 (15.8)	31 664 (14.2)
Suicide attempt^f^	194 (0.9)	1287 (1.9)	387 (1.0)	1545 (1.6)	3413 (1.5)
Other psychotropic dispensations^f^	371 (1.7)	8336 (12.2)	723 (2.0)	6878 (7.1)	16 308 (7.3)
Treatment in secondary psychiatric care^g^	2890 (13.2)	27 409 (40.2)	5929 (16.1)	34 551 (35.9)	70 779 (31.7)
Calendar year at cohort entry					
2014	4423 (20.3)	22 508 (33.0)	7067 (19.2)	32 754 (34.0)	66 752 (29.9)
2015	4356 (20.0)	14 399 (21.1)	6645 (18.0)	21 022 (21.8)	46 422 (20.8)
2016	4399 (20.2)	12 010 (17.6)	6761 (18.3)	16 765 (17.4)	39 935 (17.9)
2017	4414 (20.2)	10 523 (15.4)	7251 (19.7)	13 957 (14.5)	36 145 (16.2)
2018	4223 (19.4)	8803 (12.9)	9176 (24.9)	11 815 (12.3)	34 017 (15.2)

CMD, common mental disorder.

a. Measured during year of cohort entry.

b. Measured on 31 December in the year prior to cohort entry.

c. Measured during the calendar year prior to cohort entry.

d. Measured in the year (365 days) prior to cohort entry.

e. Measured during the entire study period (2014–2019) or up to last treatment received.

f. Measured during the year prior to, and year following, cohort entry (cohort entry date ± 365 days).

g. Measured in the four years prior to, and one year following, cohort entry (cohort entry date –1460 days to + 365 days).

### Patterns of association between patient characteristics and treatment received

Multinomial regression analyses were performed to identify patient factors associated with treatment receipt, where each treatment outcome (pharmacotherapy only, psychological therapy only or both) was compared with receiving no treatment (Table 2). Several factors consistently distinguished individuals who received no treatment from those who received any form of treatment; that is, odds ratios were in the same direction, and statistically significant, for all three pairwise comparisons (pharmacotherapy only versus no treatment; psychological therapy only versus no treatment; both versus no treatment). Specifically, when each treatment was compared with no treatment, males were less likely than females to receive pharmacotherapy (odds ratio (OR) = 0.92, 95% CI 0.89–0.95), psychological therapy (OR = 0.76, 95% CI 0.74–0.79) or both (OR = 0.77, 95% CI 0.74–0.80). Similarly, compared with Swedish-born individuals, the odds of receiving pharmacotherapy and/or psychological therapy were lower among those born in Europe (OR range = 0.86 to 0.93, 95% CI_[minimum, maximum]_ 0.80 to 0.99) and even further reduced among those born outside Europe (OR range = 0.67 to 0.70, 95% CI_[minimum, maximum]_ 0.65 to 0.73). By contrast, individuals who were single with children living at home (compared with married or cohabiting individuals without children living at home) were more likely to receive treatment (OR range = 1.11 to 1.30, 95% CI_[minimum, maximum]_ 1.03 to 1.41), as were those with >90 days sickness absence (OR range = 1.11 to 1.29, 95% CI_[minimum, maximum]_ 1.01 to 1.41). With regards to clinical factors, individuals diagnosed with depression + anxiety disorders, depression + stress-related disorders or all three disorders were more likely than those diagnosed with anxiety disorders only to receive any form of treatment (OR range = 1.25 to 29.93, 95% CI_[minimum, maximum]_ 1.14 to 37.16), whereas patients with stress-related disorders only were markedly less likely to receive treatment (OR range = 0.21 to 0.51, 95% CI_[minimum, maximum]_ 0.20 to 0.53). Treatment for comorbid mental disorders and somatic disorders in primary care also increased the likelihood of receiving any treatment (OR range = 1.27 to 1.73, 95% CI_[minimum, maximum]_ 1.20 to 1.90).

**Table 2:** Multinomial regression models, yielding odds ratios (OR) and 95% confidence intervals, examining effects of demographic, work-related and clinical factors on type of treatment received (relative to no treatment) among 223 271 individuals diagnosed with common mental disorders in primary care in Stockholm County

	Pharmacotherapy only versus no treatment (Ref)		Psychological only versus no treatment (Ref)		Both versus no treatment (Ref)	
	OR	95% CI	OR	95% CI	OR	(95% CI)
**Sociodemographic factors**						
Age (years)^a^						
20–25	Ref	–	Ref	–	Ref	–
26–35	**1**.**37**	**1.28**–**1.46**	**1**.**11**	**1.04**–**1.18**	**1**.**29**	**(1.21**–**1.37)**
36–45	**2**.**33**	**2.18**–**2.50**	0.99	0.93–1.06	**1**.**63**	**(1.52**–**1.74)**
46–55	**2**.**76**	**2.58**–**2.95**	**0**.**82**	**0.77**–**0.88**	**1**.**69**	**(1.58**–**1.80)**
56–65	**2**.**88**	**2.68**–**3.09**	**0**.**71**	**0.65**–**0.76**	**1**.**51**	**(1.41**–**1.62)**
Gender						
Female	Ref	–	Ref	–	Ref	–
Male	**0**.**92**	**0.89**–**0.95**	**0**.**76**	**0.74**–**0.79**	**0**.**77**	**(0.74**–**0.80)**
Region of birth^b^						
Sweden	Ref	–	Ref	–	Ref	–
EU25 (except Sweden)	**0**.**93**	**0.87**–**0.99**	**0**.**86**	**0.80**–**0.92**	**0**.**86**	**0.80**–**0.92**
The rest of the world	**0**.**67**	**0.65**–**0.70**	**0**.**70**	**0.67**–**0.73**	**0**.**67**	**0.65**–**0.70**
Family situation^b^						
Married or cohabiting with no children at home	Ref	–	Ref	–	Ref	–
Married or cohabiting with children at home	**0**.**94**	**0.89**–**1.00**	**1**.**12**	**1.05**–**1.20**	**1**.**07**	**1.01**–**1.14**
Single no children at home	0.99	0.94–1.04	**1**.**09**	**1.03**–**1.16**	1.04	0.99–1.10
Single with children at home	**1**.**11**	**1.03**–**1.20**	**1**.**30**	**1.19**–**1.41**	**1**.**28**	**1.19**–**1.39**
Type of living area^b^						
Big cities	Ref	–	Ref	–	Ref	–
Rural areas	0.97	0.87–1.08	**0**.**81**	**0.72**–**0.91**	1.02	0.92–1.13
Level of education^b^						
Low (0–9 years)	Ref	–	Ref	–	Ref	–
Medium (10–12 years)	**0**.**94**	**0.89**–**0.99**	**1**.**27**	**1.20**–**1.35**	**1**.**17**	**1.10**–**1.23**
High (>12 years)	1.03	0.97–1.08	**1**.**53**	**1.44**–**1.63**	**1**.**25**	**1.18**–**1.32**
**Work-related factors**						
Unemployment, days^c^						
None	Ref	–	Ref	–	Ref	–
1–180	**0**.**91**	**0.85**–**0.97**	**1**.**08**	**1.01**–**1.15**	0.96	0.90–1.03
>180	**0**.**81**	**0.73**–**0.90**	0.99	0.88–1.11	**0**.**88**	**0.79**–**0.98**
Sickness absence, days^d^						
0	Ref	–	Ref	–	Ref	–
1–90	**0**.**88**	**0.85**–**0.92**	**0**.**81**	**0.78**–**0.85**	0.99	0.95–1.03
>90	**1**.**29**	**1.19**–**1.41**	**1**.**11**	**1.01**–**1.22**	**1**.**24**	**1.14**–**1.35**
Disability pension granted^d^						
None	Ref	–	Ref	–	Ref	–
Yes	**1**.**70**	**1.54**–**1.88**	**0**.**85**	**0.75**–**0.96**	**1**.**12**	**1.01**–**1.24**
**Clinical factors**						
CMD diagnosis^e^						
Anxiety	Ref	–	Ref	–	Ref	–
Depression	**1**.**51**	**1.43**–**1.59**	1.06	1.00–1.13	**1**.**31**	**1.24**–**1.39**
Stress-related	**0**.**21**	**0.20**–**0.21**	**0**.**51**	**0.48**–**0.53**	**0**.**22**	**0.21**–**0.23**
Depression and anxiety	**5**.**09**	**4.46**–**5.81**	**3**.**05**	**2.65**–**3.51**	**13**.**09**	**11.48**–**14.93**
Depression and stress-related	**1**.**25**	**1.14**–**1.38**	**1**.**78**	**1.61**–**1.97**	**4**.**08**	**3.72**–**4.47**
Anxiety and stress-related	0.98	0.90–1.06	**2**.**04**	**1.88**–**2.21**	**3**.**61**	**3.35**–**3.90**
Depression, anxiety and stress-related	**4**.**07**	**3.26**–**5.07**	**5**.**45**	**4.35**–**6.82**	**29**.**93**	**24.11**–**37.16**
Comorbid mental disorder (any)^f^						
No	Ref	–	Ref	–	Ref	–
Yes	**1**.**70**	**1.54**–**1.88**	**1**.**46**	**1.31**–**1.62**	**1**.**73**	**1.56**–**1.90**
Comorbid somatic disorders (relevant only)^f^						
No	Ref	–	Ref	–	Ref	–
Yes	**1**.**27**	**1.20**–**1.34**	**1**.**27**	**1.20**–**1.35**	**1**.**46**	**1.38**–**1.54**
Suicide attempt^f^						
No	Ref	–	Ref	–	Ref	–
Yes	**1**.**26**	**1.07**–**1.48**	1.14	0.96–1.36	1.17	0.99–1.37
Other psychotropic dispensations^f^						
No	Ref	–	Ref	–	Ref	–
Yes	**3**.**14**	**2.80**–**3.51**	0.98	0.86–1.12	**1**.**99**	**1.78**–**2.23**
Treatment in secondary psychiatric care^g^						
No	Ref	–	Ref	–	Ref	–
Yes	**2**.**90**	**2.77**–**3.04**	1.05	1.00–1.10	**2**.**32**	**2.22**–**2.43**
Calendar year at cohort entry						
2014	Ref	–	Ref	–	Ref	–
2015	**0**.**77**	**0.74–0.81**	1.02	0.96–1.07	**0**.**81**	**0.77–0.85**
2016	**0**.**67**	**0.64–0.70**	**1**.**07**	**1.01–1.13**	**0**.**71**	**0.67–0.74**
2017	**0**.**60**	**0.57–0.63**	**1**.**17**	**1.10–1.23**	**0**.**65**	**0.62–0.68**
2018	**0**.**53**	**0.51–0.56**	**1**.**57**	**1.48–1.65**	**0**.**61**	**0.58–0.65**

Multinomial logistic regression models predicting treatment group versus reference (Ref) group; bold indicates statistical significance at *P* < 0.05 level; all variables mutually adjusted for all other variables. CMD, common mental disorder.

a. Measured during year of cohort entry.

b. Measured on 31 December in the year prior to cohort entry.

c. Measured during the calendar year prior to cohort entry.

d. Measured in the year (365 days) prior to cohort entry.

e. Measured during the entire study period (2014–2019) or up to last treatment received.

f. Measured during the year prior to, and year following, cohort entry (cohort entry date ± 365 days).

g. Measured in the four years prior to, and one year following, cohort entry (cohort entry date –1460 days to + 365 days).

### Relative contribution of patient characteristics to treatment group

Model diagnostics were used to determine the importance of each factor (Table 3). CMD diagnostic category explained the largest proportion of the variance in the model (R^2^ = 16.05%), with prior mental disorders treated in secondary care, age and calendar year at cohort entry each explaining 1–2%. Use of other psychotropic medications, receipt of disability pension, level of education, gender and region of birth made smaller contributions to the model (R^2^ range: 0.14–0.50%), whereas sickness absence, somatic comorbidities, comorbid mental disorders treated in primary care, family situation, unemployment, type of living area and suicide attempts each contributed less than 0.1%.

**Table 3:** Model diagnostics for multinomial logistic regression examining factors related to treatment receipt in 223 271 individuals diagnosed with a common mental disorder in primary care in Stockholm County

	Log-likelihood test^a^			Difference (full − reduced)^b^	
	Test statistic	d.f.	*P*-value	BIC	R^2^ (%)
Age	4511.83	12	<0.001	−4364.04	1.61
Gender	418.42	3	<0.001	−381.47	0.15
Region of birth	395.68	6	<0.001	−321.78	0.14
Family situation	116.51	9	<0.001	−5.67	0.04
Type of living area	28.95	3	<0.001	8.00	0.01
Level of education	440.56	6	<0.001	−366.67	0.16
Unemployment	65.09	6	<0.001	8.80	0.02
Sickness absence	247.21	6	<0.001	−173.31	0.09
Disability pension	493.06	3	<0.001	−456.12	0.17
CMD diagnosis	41355.43	18	<0.001	−41133.74	16.05
Comorbid mental disorder	146.10	3	<0.001	−109.16	0.05
Comorbid somatic disorder	237.21	3	<0.001	−200.26	0.08
Suicide attempts	9.46	3	0.024	27.48	0.00
Other psychotropic dispensations	1404.70	3	<0.001	−1367.75	0.50
Treatment in secondary psychiatric care	4828.21	3	<0.001	−4791.27	1.72
Calendar year at cohort entry	3384.24	12	<0.001	−3236.45	1.21

CMD, common mental disorder; BIC, Bayesian information criterion; R^2^, Nagelkerke pseudo R^2^.

a. Derived from the multinomial logistic regression. All variables mutually adjusted for all other variables.

b. Difference between full model (all variables included) and reduced model (without tested variable included). Overall values for full model: BIC: 487231.54; overall R^2^: 30.06%.

**Fig. 2:**
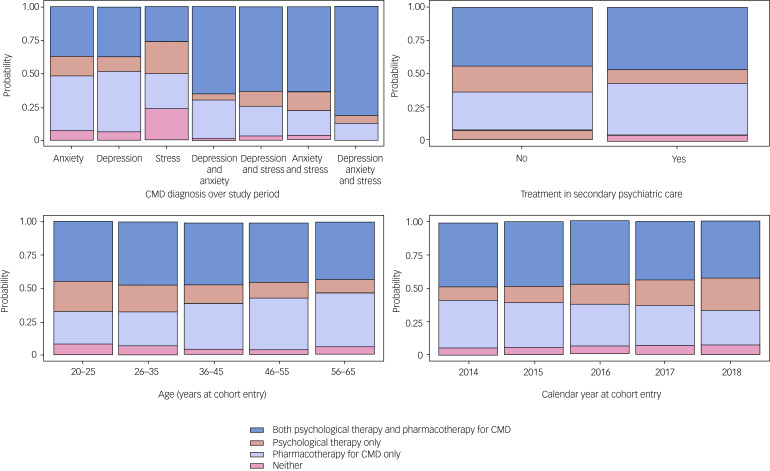
Predicted probability plots showing the likelihood of treatment group membership for predictor variables with Nagelkerke R^2^ values >1%.

Predicted probability plots for predictors with R^2^ values exceeding 1% are shown in Fig. 2 to illustrate the associations between these factors and treatment receipt. Except for the CMD diagnostic category (panel A), these predictors were associated with the relative likelihood of receiving pharmacotherapy only versus psychological therapy only, rather than the likelihood of receiving none versus any treatment. Specifically, the relative probability of receiving psychological therapy only was lower among those previously treated in secondary care (panel B) and decreased with age (panel C) but was higher among those who entered the cohort in later years (panel D). For the CMD diagnostic category, groups differed in their likelihood of receiving no treatment (highest among those with stress-related disorders only) or both treatments (highest among those with all three diagnoses) as well as their relative probability of receiving pharmacotherapy only (highest among those with depression only) or psychological therapy only (highest among those with stress-related disorders only).

## Discussion

More than 90% of a large, contemporaneous cohort of individuals diagnosed with CMDs in Stockholm primary care clinics received some form of treatment proximal to their diagnosis, most commonly a combination of pharmacotherapy and psychological therapy. Several factors, including gender, region of birth, long-term sickness absence, living status, CMD diagnostic group and mental and somatic comorbidities diagnosed in primary care, were found to consistently distinguish those who received no treatment from those who received either or both. CMD diagnostic group made the largest contribution to the model; the next highest-ranking variables (prior treatment in secondary psychiatric care, age and calendar year at cohort entry) tended to be associated with relative probabilities of pharmacotherapy or psychological therapy only.

Previous studies have reported highly variable rates of treatment receipt among individuals with CMDs in primary care, ranging from 36 to 82%.^23–28^ The high treatment rate that we observed in this sample probably reflects the fact that we were able to capture psychological therapies as well as medication dispensations. Indeed, the proportion of patients in the current study who received pharmacotherapy (73.7%), either alone or in combination with psychological treatments, is largely consistent with those reported by other European register-based studies.^23–25^ Moreover, we employed a broad time-frame for measuring treatment receipt, including treatments received up to 1 month before and 12 months after any diagnosis of CMD. Our finding that individuals with CMDs were more likely to receive pharmacotherapy than psychological therapy is consistent with community-based surveys^13^ but is at odds with patient preference studies, which show that 75% of individuals with CMDs would prefer to receive psychological treatment.^47^ Notably, we found that the relative probability of receiving psychological therapy in isolation increased in parallel with calendar year at cohort entry; this trend could reflect increased availability of psychological therapies over time or indicate that patients showed a greater preference for psychological therapy in later years. Importantly, it was most common for patients in our sample to receive both treatment modalities: given that combined treatment (psychotherapy and pharmacotherapy) is estimated to be around twice as effective as pharmacotherapy alone in treating major depression, panic disorder and obsessive–compulsive disorder,^48^ these findings are particularly reassuring and suggest that a substantial proportion of patients treated in primary care receive the most effective treatment package.

The strongest predictors of type of treatment received were clinical factors, most notably CMD diagnostic category and treatment in secondary psychiatric care. Individuals with disorders in all three diagnostic categories had the highest probability of receiving both treatment types, probably reflecting the fact that this group had the most complex clinical presentations. One novel finding was that individuals with stress-related disorders had the lowest probability of receiving any treatment. Indeed, around 60% of patients who did not receive any treatment presented with a stress-related disorder only. This may be explained by the fact that the most common diagnosis within this category was acute stress disorder, which is considered in the most recent ICD revision (ICD-11)^49^ to be a normal response to an extreme stressor rather than a mental disorder.^50^ Given these classification changes, we might not expect individuals presenting with acute stress reactions in the absence of comorbid depression to be offered psychological or pharmacological therapy. Our finding that individuals who were treated in secondary psychiatric care in the 4 years prior to and 1 year after cohort entry were more likely than those who were not to receive pharmacotherapy alone and were particularly unlikely to receive psychological therapy alone was perhaps unsurprising. Patients who have experienced psychiatric disorders that are sufficiently severe as to warrant treatment in secondary care might be managed differently by primary care clinicians, who may, for example, be less willing to risk placing the patient on a waiting list for psychological therapy and/or might be more inclined to prescribe medication that was commenced in secondary care. Consistent with the findings for clinical factors, we observed that that individuals with sickness absence >90 days were more likely to receive treatment, suggesting that functional impairment leading to long-term work disability is associated with higher treatment intensity.

Among the sociodemographic factors examined, patient age explained the largest proportion of variance in treatment received. Although we found no evidence to suggest that individuals in a particular age group were less likely to receive any treatment, the type of treatment received differed across age groups. Specifically, the relative probability of receiving pharmacotherapy alone increased with age, coinciding with a decrease in the probability of receiving psychotherapy only. Partially consistent with these findings, a recent US study of primary care patients with depression observed that the odds of receiving any treatment decreased with age and that among those who had initiated treatment, the likelihood of receiving psychological therapy (rather than pharmacotherapy) was also reduced in older age groups.^27^ Similarly, a UK survey found that older adults (aged 75+ years) in the general population were less likely than younger adults to receive ‘talking therapy’,^21^ and a study examining access to psychological therapies among individuals with anxiety disorders in Canadian primary care clinics found that those aged >60 years were less likely than those aged 25–44 to receive treatment.^26^ These findings could reflect barriers to accessing and utilising psychological treatments among older adult populations, although patient preference studies indicate that younger samples are more likely to prefer psychological treatment than older samples.^47^

Consistent with a recent UK population survey,^16^ we observed that males and individuals born outside Sweden (particularly those born outside Europe) were less likely to receive any form of treatment. Primary care studies, however, have reported conflicting findings with respect to gender, with some studies reporting higher^27^ and lower^26^ rates of treatment in males relative to females and other studies observing no differences.^28,29^ Although primary care studies conducted in the US have found that ethnic minority groups are less likely to receive treatment than their non-Hispanic White peers,^27,29^ studies examining migrant status are lacking. Register-based studies have shown that migrants are less likely to use mental health services and use psychotropic medications, irrespective of diagnosis, despite being more likely to develop psychiatric disorders.^51^ Cultural factors and (particularly for psychological therapy) language differences may contribute to under-utilisation of treatments among migrants with CMDs.

### Limitations

Owing to our use of the extensive national registers in Sweden, our study had the advantages of a large sample size and access to a wide range of sociodemographic, work-related and clinical factors. Moreover, the comprehensive coverage of Region Stockholm's VAL database meant that we were able to identify patients with CMDs treated in public primary care clinics and the majority of private clinics. However, some limitations should be noted. First, although we can be confident that the PDR captures all prescribed and dispensed medications, we cannot be certain that every session of psychological therapy is recorded in VAL. However, given that ~100% of all primary care clinics in Sweden have electronic data capture systems^52^ designed to facilitate reliable, high-quality data recording, it is highly likely that all individuals treated with psychological therapy had at least one session recorded. Moreover, any misclassification can be assumed to be non-differential. Second, as we did not examine treatment dose, we cannot say that individuals with CMDs received optimal or minimally adequate doses of pharmacotherapy or psychological therapy. However, we intended to investigate the factors associated with psychological and pharmacological therapy receipt, irrespective of whether patients persisted with these treatments. Finally, our findings may have limited generalisability to non-European countries without publicly funded healthcare.^32^ Indeed, given that primary care clinicians in Sweden are required to undertake mental health training as part of their continuing professional development,^52^ detection and adequate treatment of CMDs may be better than in countries where this is not required.

### Future perspectives

We observed that the vast majority of individuals in primary care received pharmacological and/or psychological therapy proximal to a CMD diagnosis. Although our findings indicate that individuals with the highest illness burden (i.e. those with multiple CMD diagnoses, long-term sickness absence and mental/somatic comorbidities) received the most intensive treatments, even after adjusting for these factors, males and individuals born outside Sweden were less likely to receive any form of therapy. These findings, which are partially consistent with those of studies conducted in other countries, highlight important treatment disparities that exist even in a well-resourced European country. Further research investigating the complex factors that contribute to the provision and uptake of psychological and pharmacological therapies may help to ensure more equitable treatment of CMDs in primary care settings. Of note, further studies examining treatment received during the COVID-19 pandemic might generate a different picture, given that primary care has developed a range of digital solutions for providing treatments that might enable better outreach.

## Data Availability

The data used in this study cannot be made publicly available owing to privacy regulations. According to the General Data Protection Regulation, the Swedish law SFS 2018:218, the Swedish Data Protection Act, the Swedish Ethical Review Act and the Public Access to Information and Secrecy Act, these types of sensitive data can only be made available for specific purposes, including research, that meet the criteria for access to this type of sensitive and confidential data as determined by a legal review. Readers may contact K.A. (kristina.alexanderson@ki.se) regarding the data.

## Supplementary material

For supplementary material accompanying this paper visit http://doi.org/10.1192/bjo.2023.8. click here to view supplementary material
