# Supplementary material for: Patient factors associated with receipt of psychological and pharmacological treatments among individuals with common mental disorders in a Swedish primary care setting
Source: BJPsych Open. 2023 Feb 28;9(2):e40. doi: 10.1192/bjo.2023.8 (PMC10044006; doi:10.1192/bjo.2023.8)
Supplement: Supplementary file 1 [file S205647242300008Xsup001.docx]

Supplementary Material

## Psychological therapy in primary care

Receipt of psychological therapy in primary care was determined using Region Stockholm’s VAL database (VAL). All visits to primary care recorded within this database are coded according to:

- Date of visit
- Diagnosis (1-8 per visit) coded according to the International Classification of Diseases – version 10 (ICD-10)
- Action code (1-10 per visit), defined according to the Swedish classification of care measures (KVÅ: <https://www.socialstyrelsen.se/utveckla-verksamhet/e-halsa/klassificering-och-koder/kva/>)
- Healthcare provider (1 to 5 per visit)
- Type of contact (1 per visit), including information on whether the visit occurred

We employed a broad definition of psychological therapy that included (i) any visit that with an action code that corresponded to a systematic psychological therapy (see Table S1) or ‘Curator’, or (ii) any other visit (irrespective of action code) where the healthcare provider was a psychologist, psychotherapist, or ‘curator’. For both, we excluded events where no patient contact occurred (e.g., a visit was scheduled but the patient did not attend) as determined using the ‘type of contact’ variable. If a visit included more than one relevant action code/caregiver type, each was code separately.

**Table S1** Psychological therapy action codes

| Action code | Treatment type |
| --- | --- |
| DU008 | Systematic psychological treatment, psychodynamic (PDT) |
| DU009 | Systematic psychological treatment, other |
| DU010 | Systematic psychological treatment, cognitive |
| DU011 | Systematic psychological treatment, cognitive behavioural therapy (CBT) |
| DU013 | Systematic psychological treatment, mentalization based (MBT) |
| DU014 | Eye movement desensitisation reprogramming (EMDR) |
| DU020 | Systematic psychological treatment, systemic |
| DU021 | Systematic psychological treatment, dialectical behaviour therapy (DBT) |
| DU022 | Systematic psychological treatment, interpersonal therapy (IPT) |
| XS913 | Curator |

**Table S2** Types of pharmacological therapy received in a sample of 223,271 individuals diagnosed with common mental disorders in primary care in Stockholm County

|  | Pharmacotherapy only (N=68,243) | | Both pharmacotherapy and psychological therapy  (N=96,313) | | Total with pharmacotherapy  (N= 164,556) | |
| --- | --- | --- | --- | --- | --- | --- |
| **Medication class (ATC-code)** | **N** | **(%)** | **N** | **(%)** | **N** | **(%)** |
| Antidepressants (N06A) | 50,521 | (74.0) | 74,565 | (77.4) | 125,086 | (76.0) |
| Anxiolytics (N05B) | 33,114 | (48.5) | 55,041 | (57.1) | 88,155 | (53.6) |
| Hypnotics/sedatives (N05C/R06AD01) | 32,941 | (48.3) | 50,985 | (52.9) | 83,926 | (51.0) |

ATC: Anatomical Therapeutic Chemical. Medication classes are not mutually exclusive (individuals could receive more than one type of medication within the study period). Only prescription dispensations that occurred proximal to a CMD diagnosis were included.

**Table S3** Types of psychological therapy received in 223,271 individuals diagnosed with common mental disorders in primary care in Stockholm County

|  | Psychological therapy only (N=36,900) | | Both psychological therapy and pharmacotherapy  (N=96,313) | | Total with psychological therapy  (N=133,213) | |
| --- | --- | --- | --- | --- | --- | --- |
| **Psychological therapy** | **N** | **(%)** | **N** | **(%)** | **N** | **(%)** |
| Systematic psychological therapy | 18,474 | (50.1) | 49,191 | (51.1) | 67,665 | (50.8) |
| Psychodynamic therapy | 1,611 | (4.4) | 4,767 | (4.9) | 6,378 | (4.8) |
| Other systematic psychological therapy | 427 | (1.2) | 1,408 | (1.5) | 1,835 | (1.4) |
| Cognitive therapy | 220 | (0.6) | 755 | (0.8) | 975 | (0.7) |
| CBT | 16,694 | (45.2) | 44,651 | (46.4) | 61,345 | (46.1) |
| MBT | 36 | (0.1) | 132 | (0.1) | 168 | (0.1) |
| EDMR | 14 | (0.0) | 35 | (0.0) | 49 | (0.0) |
| Systemic therapy | 44 | (0.1) | 87 | (0.1) | 131 | (0.1) |
| DBT | <10 | (0.0) | 22 | (0.0) | 28 | (0.0) |
| IPT | 541 | (1.5) | 1,522 | (1.6) | 2,063 | (1.5) |
| Other psychological therapy | 18,426 | (49.9) | 47,122 | (48.9) | 65,548 | (49.2) |
| Psychologist | 8,166 | (22.1) | 21,248 | (22.1) | 29,414 | (22.1) |
| Curator | 8,505 | (23.0) | 23,731 | (24.6) | 32,236 | (24.2) |
| Psychotherapist | 3,558 | (9.6) | 9,640 | (10.0) | 13,198 | (9.9) |

CBT: cognitive behavioural therapy; MBT: mentalization based therapy; EMDR: Eye movement desensitisation reprogramming; DBT: dialectical behavioural therapy; IPT: interpersonal therapy. Psychological therapy classes are not mutually exclusive (individuals could receive more than one type of therapy within the study period). Only psychological therapy visits that occurred proximal to a CMD diagnosis were included.

**Table S4** Region of birth among 223,271 individuals diagnosed with common mental disorders in primary care in Stockholm County

|  | No treatment  (N=21,815, 9.8%) | | Pharmacotherapy only (N=68,243, 30.6%) | | Psychological therapy only  (N=36,900, 16.5%) | | Both  (N=96,313, 43.1%) | | Total sample  (N=223,271) | |
| --- | --- | --- | --- | --- | --- | --- | --- | --- | --- | --- |
| **Region of birth** | **N** | **(%)** | **N** | **(%)** | **N** | **(%)** | **N** | **(%)** | **N** | **(%)** |
| Afghanistan | 93 | (0.4) | 307 | (0.4) | 123 | (0.3) | 365 | (0.4) | 888 | (0.4) |
| Africa (excluding Eritrea, Ethiopia, and Somalia) | 410 | (1.9) | 859 | (1.3) | 475 | (1.3) | 1,232 | (1.3) | 2,976 | (1.3) |
| Asia (excluding Afghanistan, Iraq, Iran, and Syria) | 1,183 | (5.4) | 3,019 | (4.4) | 1,506 | (4.1) | 4,222 | (4.4) | 9,930 | (4.4) |
| Chile | 222 | (1.0) | 832 | (1.2) | 349 | (0.9) | 1,261 | (1.3) | 2,664 | (1.2) |
| Eritrea | 115 | (0.5) | 148 | (0.2) | 122 | (0.3) | 198 | (0.2) | 583 | (0.3) |
| Ethiopia | 173 | (0.8) | 208 | (0.3) | 137 | (0.4) | 268 | (0.3) | 786 | (0.4) |
| EU15 (excluding Denmark, Finland, and Sweden | 396 | (1.8) | 1,083 | (1.6) | 664 | (1.8) | 1,481 | (1.5) | 3,624 | (1.6) |
| Europe (excluding EU15, the Nordic countries, and the former Yugoslavia) | 700 | (3.2) | 2,271 | (3.3) | 958 | (2.6) | 2,893 | (3.0) | 6,822 | (3.1) |
| Finland | 422 | (1.9) | 1697 | (2.5) | 583 | (1.6) | 1,771 | (1.8) | 4,473 | (2.0) |
| former Yugoslavia | 320 | (1.5) | 1,011 | (1.5) | 404 | (1.1) | 1,295 | (1.3) | 3,030 | (1.4) |
| Iran | 427 | (2.0) | 1,814 | (2.7) | 752 | (2.0) | 2,676 | (2.8) | 5,669 | (2.5) |
| Iraq | 781 | (3.6) | 1,712 | (2.5) | 854 | (2.3) | 2,396 | (2.5) | 5,743 | (2.6) |
| North America | 113 | (0.5) | 510 | (0.7) | 244 | (0.7) | 643 | (0.7) | 1,510 | (0.7) |
| Oceania | 19 | (0.1) | 62 | (0.1) | 30 | (0.1) | 75 | (0.1) | 186 | (0.1) |
| Somalia | 103 | (0.5) | 169 | (0.2) | 104 | (0.3) | 189 | (0.2) | 565 | (0.3) |
| South America (excluding Chile) | 321 | (1.5) | 729 | (1.1) | 504 | (1.4) | 1,198 | (1.2) | 2,752 | (1.2) |
| Soviet Union | 37 | (0.2) | 113 | (0.2) | 41 | (0.1) | 152 | (0.2) | 343 | (0.2) |
| Sweden | 15,599 | (71.5) | 50,768 | (74.4) | 28,614 | (77.5) | 72,885 | (75.7) | 167,866 | (75.2) |
| Syria | 236 | (1.1) | 546 | (0.8) | 243 | (0.7) | 622 | (0.6) | 1,647 | (0.7) |
| The Nordic countries (excluding Sweden and Finland) | 138 | (0.6) | 379 | (0.6) | 189 | (0.5) | 484 | (0.5) | 1,190 | (0.5) |

Detailed information on region of birth is obtained from Longitudinal Database for Integration Studies (STATIV) register, available from 1997-2016, missing data (n=24).

**Table S5** Prevalence of specific common mental disorder diagnoses in 223,271 individuals diagnosed with common mental disorders in primary care in Stockholm County

|  | No treatment  (N=21,815, 9.8%) | | Pharmacotherapy only (N=68,243, 30.6%) | | Psychological therapy only  (N=36,900, 16.5%) | | Both  (N=96,313, 43.1%) | | Total sample  (N=223,271) | |
| --- | --- | --- | --- | --- | --- | --- | --- | --- | --- | --- |
| **Disorder (ICD-10 code)** | **N** | **(%)** | **N** | **(%)** | **N** | **(%)** | **N** | **(%)** | **N** | **(%)** |
| Depressive disorders, any (F32-F39) | 3,105 | (14.2) | 30,163 | (44.2) | 9,433 | (25.6) | 51,461 | (53.4) | 94,162 | (42.2) |
| Depressive episode (F32) | 2,669 | (12.2) | 22,908 | (33.6) | 8,102 | (22.0) | 43,727 | (45.4) | 77,406 | (34.7) |
| Recurrent depressive disorder (F33) | 339 | (1.6) | 9,561 | (14.0) | 1,194 | (3.2) | 14,714 | (15.3) | 25,808 | (11.6) |
| Persistent mood disorders (F34) | 59 | (0.3) | 489 | (0.7) | 204 | (0.6) | 765 | (0.8) | 1,517 | (0.7) |
| Other mood disorders (F38) | <10 | (0.0) | 41 | (0.1) | 27 | (0.1) | 78 | (0.1) | 152 | (0.1) |
| Unspecified mood disorder (F39) | 88 | (0.4) | 477 | (0.7) | 318 | (0.9) | 865 | (0.9) | 1,748 | (0.8) |
| Anxiety disorders, any (F40-F42) | 5,998 | (27.5) | 35,348 | (51.8) | 17,103 | (46.3) | 61,825 | (64.2) | 120,274 | (53.9) |
| Phobic anxiety disorders (F40) | 368 | (1.7) | 2,430 | (3.6) | 972 | (2.6) | 3,808 | (4.0) | 7,578 | (3.4) |
| Other anxiety disorders (F41) | 5,602 | (25.7) | 33,517 | (49.1) | 16,439 | (44.6) | 60,615 | (62.9) | 116,173 | (52.0) |
| Obsessive-compulsive disorder (F42) | 135 | (0.6) | 1,122 | (1.6) | 370 | (1.0) | 1,569 | (1.6) | 3,196 | (1.4) |
| Stress-related disorders, any (F43) | 14,586 | (66.9) | 23,661 | (34.7) | 20,756 | (56.2) | 51,341 | (53.3) | 110,344 | (49.4) |
| Acute stress reaction (F43.0) | 8,168 | (37.4) | 11,754 | (17.2) | 10,052 | (27.2) | 25,162 | (26.1) | 55,136 | (24.7) |
| Post-traumatic stress disorder (F43.1) | 243 | (1.1) | 1,805 | (2.6) | 556 | (1.5) | 3,316 | (3.4) | 5,920 | (2.7) |
| Adjustment disorders (F43.2) | 684 | (3.1) | 1,143 | (1.7) | 1,978 | (5.4) | 4,402 | (4.6) | 8,207 | (3.7) |
| Exhaustion disorder (F43.8A) | 2,462 | (11.3) | 6,352 | (9.3) | 4,873 | (13.2) | 19,175 | (19.9) | 32,862 | (14.7) |
| Other reactions to severe stress (F43.8) | 3,654 | (16.8) | 7,888 | (11.6) | 6,827 | (18.5) | 22,967 | (23.8) | 41,336 | (18.5) |
| Reaction to severe stress, unspecified (F43.9) | 3,219 | (14.8) | 5,302 | (7.8) | 6,735 | (18.3) | 15,628 | (16.2) | 30,884 | (13.8) |

ICD-10: International Classification of Diseases – version 10. Disorder categories are not mutually exclusive. For individuals who received neither pharmacotherapy no psychological therapy, all diagnoses from 2014-2019 are included; for individuals who received pharmacotherapy, psychological therapy, both, all diagnoses recorded up to date of last observed treatment are included.

**Table S5** Comorbid mental disorders diagnosed in primary care in the year prior to and year after cohort entry among 223,271 individuals diagnosed with common mental disorders in primary care in Stockholm County

|  | No treatment  (N=21,815, 9.8%) | | Pharmacotherapy only  (N=68,243, 30.6%) | | Psychological therapy only  (N=36,900, 16.5%) | | Both  (N=96,313, 43.1%) | | Total sample  (N=223,271) | |
| --- | --- | --- | --- | --- | --- | --- | --- | --- | --- | --- |
| **Disorder (ICD-10 code)** | **N** | **(%)** | **N** | **(%)** | **N** | **(%)** | **N** | **(%)** | **N** | **(%)** |
| Organic mental disorders (F00-F09) | 13 | (0.1) | 34 | (0.1) | 99 | (0.1) | 127 | (0.1) | 273 | (0.1) |
| Substance use disorders (F10-F19) | 91 | (0.4) | 161 | (0.4) | 1,332 | (2.0) | 1,151 | (1.2) | 2,735 | (1.2) |
| Psychotic disorders (F20-F29) | <10 | (0.0) | <10 | (0.0) | 37 | (0.1) | 36 | (0.0) | 83 | (0.0) |
| Bipolar affective disorder (F30-F31) | <10 | (0.0) | <10 | (0.0) | 89 | (0.1) | 105 | (0.1) | 206 | (0.1) |
| Dissociative, somatoform and other neurotic disorders (F44-F48) | 28 | (0.1) | 122 | (0.3) | 279 | (0.4) | 398 | (0.4) | 827 | (0.4) |
| Behavioural syndromes associated with physiological disturbances/factors (F50-F59) | 140 | (0.6) | 350 | (0.9) | 1,472 | (2.2) | 2,307 | (2.4) | 4,269 | (1.9) |
| Personality disorders (F60-F69) | 34 | (0.2) | 73 | (0.2) | 365 | (0.5) | 348 | (0.4) | 820 | (0.4) |
| Intellectual disabilities (F70-F79) | 26 | (0.1) | 17 | (0.0) | 182 | (0.3) | 78 | (0.1) | 303 | (0.1) |
| Developmental disorders (F80-F89) | 91 | (0.4) | 155 | (0.4) | 720 | (1.1) | 563 | (0.6) | 1,529 | (0.7) |
| Behavioural/emotional disorders with onset in childhood and adolescence (F90-F98) | 115 | (0.5) | 313 | (0.8) | 1,491 | (2.2) | 1,393 | (1.4) | 3,312 | (1.5) |
| Unspecified mental disorder (F99) | 17 | (0.1) | 251 | (0.7) | 136 | (0.2) | 633 | (0.7) | 1,037 | (0.5) |

ICD-10: International Classification of Diseases – version 10. Disorder categories are not mutually exclusive and include all disorders recorded in primary care in the year prior to, and the year after, cohort entry date.

**Table S6** Mental disorders treated in secondary care in the four years prior to, and year of cohort entry, among 223,271 individuals diagnosed with common mental disorders in primary care in Stockholm County

|  | No treatment  (N=21,815, 9.8%) | | Pharmacotherapy only (N=68,243, 30.6%) | | Psychological therapy only  (N=36,900, 16.5%) | | Both  (N=96,313, 43.1%) | | Total sample  (N=223,271) | |
| --- | --- | --- | --- | --- | --- | --- | --- | --- | --- | --- |
| **Disorder (ICD-10 code)** | **N** | **(%)** | **N** | **(%)** | **N** | **(%)** | **N** | **(%)** | **N** | **(%)** |
| Organic mental disorders (F00-F09) | <10 | (0.0) | 131 | (0.2) | 18 | (0.0) | 127 | (0.1) | 285 | (0.1) |
| Substance use disorders (F10-F19) | 459 | (2.1) | 5,495 | (8.1) | 804 | (2.2) | 5,040 | (5.2) | 11,798 | (5.3) |
| Psychotic disorders (F20-F29) | 11 | (0.1) | 194 | (0.3) | 16 | (0.0) | 145 | (0.2) | 366 | (0.2) |
| Bipolar affective disorder (F30-F31) | 18 | (0.1) | 532 | (0.8) | 35 | (0.1) | 490 | (0.5) | 1,075 | (0.5) |
| Depressive disorders (F32-F39) | 532 | (2.4) | 14,186 | (20.8) | 1,332 | (3.6) | 15,936 | (16.5) | 31,986 | (14.3) |
| Anxiety disorders (F40-F42) | 937 | (4.3) | 14,176 | (20.8) | 2,108 | (5.7) | 16,438 | (17.1) | 33,659 | (15.1) |
| Stress-related disorders (F43) | 948 | (4.3) | 7,709 | (11.3) | 2,003 | (5.4) | 10,976 | (11.4) | 21,636 | (9.7) |
| Dissociative, somatoform and other neurotic disorders (F44-F48) | 146 | (0.7) | 1,007 | (1.5) | 309 | (0.8) | 1,208 | (1.3) | 2,670 | (1.2) |
| Behavioural syndromes associated with physiological disturbances/factors (F50-F59) | 128 | (0.6) | 1,619 | (2.4) | 353 | (1.0) | 1,840 | (1.9) | 3,940 | (1.8) |
| Personality disorders (F60-F69) | 110 | (0.5) | 1,866 | (2.7) | 199 | (0.5) | 1,426 | (1.5) | 3,601 | (1.6) |
| Intellectual disabilities (F70-F79) | 22 | (0.1) | 288 | (0.4) | 18 | (0.0) | 129 | (0.1) | 457 | (0.2) |
| Developmental disorders (F80-F89) | 95 | (0.4) | 1,223 | (1.8) | 114 | (0.3) | 718 | (0.7) | 2,150 | (1.0) |
| Behavioural/emotional disorders with onset in childhood and adolescence (F90-F98) | 284 | (1.3) | 3,443 | (5.0) | 566 | (1.5) | 2,646 | (2.7) | 6,939 | (3.1) |
| Unspecified mental disorder (F99) | 24 | (0.1) | 312 | (0.5) | 73 | (0.2) | 409 | (0.4) | 818 | (0.4) |

ICD-10: International Classification of Diseases – version 10. Disorder categories are not mutually exclusive and include all disorders recorded in secondary care in the four years prior to, and the year after, cohort entry date.
